# Legal Responsibilitg of Discontinuing Medical Attendance

**Published:** 1848-06

**Authors:** 


*Legal responsibility of discontinuing medical attendance.*—A legal case lately occurred in the city of New-York, which involves a point of some interest to the Profession. To compel a Physician, *nolens volens*, to continue his medical attendance in a case from which he wishes to withdraw, would, certainly, be an outrageous invasion of the rights of personal freedom. We can see, however, nothing unreasonable in requiring that he give due notice of his intention to withhold his services. We suspect this is the first instance in which this question has come up for legal adjustment. Now that a precedent is established, such is the itching with some persons to obtain legal redress for supposed medical grievences, it behooves medical men to be on their guard against taking *French leave* of their patients.—We have only to say 'this is my last visit,' and the requisition of the law is satisfied. We take the following account of the suit referred to from the Annalist:—

"A suit was recently brought in this city against Dr. Peter Pratt, by a patient, who accused him of having abruptly ceased in his attendance after calling a few times, in consequence of which delay the health of the plaintiff was impaired, &c. The gist of the matter seems to be, that the Doctor did not announce to the patient his intention to discontinue his attendance; and the defence was, that the patient had resorted to other medicines than those ordered. The damages were laid at $5000. In all such cases the patient should undoubtedly be informed of the physicians intention. But we think, that finding his patient intractable and indisposed to follow his directions, and willing to listen rather to the suggestions of his own judgment, or the mischievous advice of ignorant and meddlesome intruders, Dr. Pratt was perfectly justified in withdrawing from the case—was, in fact, bound to do so, and we sincerely hope that every other physician will promptly and unhesitatingly in like cases do the same. The public must be taught a few lessons, and the sooner they are inculcated the better. Physicians *must*, if they would make it respectable, stand up for the dignity of their calling, irrespective of pecuniary considerations. Judge Ulshoeffer's charge appears to us very sensible and proper, and we reccommend it to the attention of our readers. "A physician, when once employed to attend a patient, cannot afterwards withdraw himself, without giving due notice to the patient, so as the latter might provide himself with another physician; but by giving such notice, he is at liberty to withdraw from his attendance, and the patient cannot maintain an action. If a patient neglects to comply with the directions of his physician, and does not take his prescriptions, the physician may withdraw without subjecting himself to an action at law; and lastly, that if an injury arises to a patient from the combined neglect of himself and his physician, in such case the patient cannot maintain an action." The jury gave the plaintiff a verdict of six cents damages. The facts of the case, we think, should be given authentically to the profession·

